# Association of hypercalciuria with vitamin D supplementation in patients undergoing ketogenic dietary therapy

**DOI:** 10.3389/fnut.2022.970467

**Published:** 2022-09-02

**Authors:** Myeongseob Lee, Hae In Lee, Kyungchul Song, Han Saem Choi, Junghwan Suh, Se Hee Kim, Hyun Wook Chae, Hoon-Chul Kang, Joon Soo Lee, Heung Dong Kim, Ho-Seong Kim, Ahreum Kwon

**Affiliations:** ^1^Department of Pediatrics, Severance Children’s Hospital, Endocrine Research Institute, Yonsei University College of Medicine, Seoul, South Korea; ^2^Department of Pediatrics, CHA Gangnam Medical Center, CHA University, Seoul, South Korea; ^3^Department of Pediatrics, International St. Mary’s Hospital, Catholic Kwandong University, Incheon, South Korea; ^4^Division of Pediatric Neurology, Department of Pediatrics, Severance Children’s Hospital, Yonsei University College of Medicine, Seoul, South Korea

**Keywords:** vitamin D, vitamin D deficiency, ketogenic diet, hypercalciuria, urolithiasis

## Abstract

**Background:**

Ketogenic dietary therapy (KDT) is used as an effective treatment for epilepsy. However, KDT carries the risk of bone health deterioration; therefore, vitamin D supplementation is required. Vitamin D replacement therapy in KDT has not been established because it may be related to hypercalciuria/urolithiasis, which are common adverse effects of KDT. Hence, this study aimed to evaluate the dose-dependent association between vitamin D_3_ and hypercalciuria/urolithiasis in patients undergoing KDT and dose optimization for renal complications.

**Materials and methods:**

Overall, 140 patients with intractable childhood epilepsy started 3:1 KDT (lipid to non-lipid ratio) at the Severance Children’s Hospital from January 2016 to December 2019. Regular visits were recommended after KDT initiation. Participants were assessed for height, weight, serum 25-hydroxyvitamin D (25-OH-D_3_) level, parathyroid hormone level, and ratio of urinary excretion of calcium and creatinine (Uca/Ucr). Kidney sonography was conducted annually. Patients who already had urolithiasis and were taking hydrochlorothiazide before KDT, failed to maintain KDT for 3 months, did not visit the pediatric endocrine department regularly, did not take prescribed calcium and vitamin D3 properly, or needed hospitalization for > 1°month because of serious medical illness were excluded. Data from patients who started diuretic agents, e.g., hydrochlorothiazide, were excluded from that point because the excretion of calcium in the urine may be altered in these patients.

**Result:**

In total, 49 patients were included in this study. Uca/Ucr ratio significantly decreased with increasing levels of 25-OH-D_3_ (*p* = 0.027). The odds ratio for hypercalciuria was 0.945 (95% confidence interval, 0.912–0.979; *p* = 0.002) per 1.0 ng/mL increment in 25-OH-D_3_ level. Based on findings of receiver operating characteristic curve analysis and Youden’s J statistic, the cut-off 25-OH-D_3_ level for preventing hypercalciuria was > 39.1 ng/mL at 6 months. Furthermore, the vitamin D_3_ supplementation dose cut-off was > 49.5 IU/kg for hypercalciuria prevention.

**Conclusion:**

An inverse relationship between Uca/Ucr ratio and 25-OH-D_3_ level was noted, which means that vitamin D supplementation is helpful for preventing hypercalciuria related to KDT. We suggest that the recommended 25-OH-D_3_ level is > 40 ng/mL for hypercalciuria prevention and that KDT for children with epilepsy can be optimized by vitamin D_3_ supplementation at 50 IU/kg.

## Introduction

Since 1921, ketogenic dietary therapy (KDT) has been considered a well-known non-pharmacologic anti-convulsant treatment for both children and adults with multi-drug resistant epilepsy (1). KDT is based on the fact that lipophilic compounds known as ketone bodies, such as acetoacetate, acetone, and beta-hydroxybutyrate, can cross the blood–brain barrier and act as direct anticonvulsants (2). It is also known that intermediate chain triglycerides, such as decanoic acid, can directly inhibit α-amino-3-hydroxy-5-methyl-4-isoxazolepropionic acid (AMPA) receptors, and that KDT increases adenosine level and inhibits DNA methylation, which are the known key mechanisms of KDT for treating epilepsy (3). However, KDT without careful management may accompany various side effects such as gastrointestinal symptoms, hepatic dysfunction, dyslipidemia, growth retardation, urolithiasis, pancreatitis, and cardiac abnormalities, especially when it is used together with high-dose anti-epileptic drugs (AEDs) (1).

Bone health deterioration is one of the most common clinical issues in patients undergoing KDT. Patients with intractable epilepsy usually have prolonged exposure to high-dose AEDs and are at risk of vitamin D deficiency (4). Furthermore, KDT is a diet prone to being deficient in essential nutrients, including calcium, phosphorus, magnesium, vitamin K, and vitamin D. Additionally, ketone bodies produced by KDT also induce acidification, which converts active vitamin D into an inactive form (1). Therefore, KDT can lead to calcium and vitamin D deficiencies and worsen bone vulnerability, causing osteoporosis; hence, calcium and vitamin D supplementation are needed. Given the possible risks and complications of KDT, patients on KDT are advised to visit an endocrinologist regularly to ensure adequate calcium and vitamin D supplementation.

Meanwhile, hypercalciuria and urolithiasis are also common complications of KDT, which are caused by increased bone demineralization due to acidosis. Acidosis induces hypocitraturia, which in turn increases free calcium, and increases the less soluble uric acid levels (5). As calcium is the most frequent component of urinary calculi and the major constituent of approximately 75% of the stones, an increase in the urinary excretion of calcium is the most common risk factor for urolithiasis (6, 7). Low urine volume and hypercalciuria increase Randall’s plaque formation, which is specific to calcium oxalate stone formation (8, 9). Consequently, urolithiasis may develop in children undergoing KDT (10, 11). Although hypercalciuria and urolithiasis are not absolute contraindications for KDT or indications for cessation of KDT (1), they may cause poor compliance and treatment failure, as the presence of kidney stones may lead to severe abdominal pain or dysuria, which may reduce the patient’s quality of life.

Calcium intake and vitamin D supplementation have been thought to be risk factors for hypercalciuria because they can increase intestinal absorption of calcium and cause hypercalcemia, even without KDT (12). However, as mentioned above, calcium and vitamin D supplementation are required for patients undergoing KDT. In addition, as vitamin D levels in patients with urolithiasis are lower than those in the normal population, vitamin D deficiency is thought to increase the occurrence of kidney stone formation (13). Therefore, whether calcium intake and vitamin D supplementation worsen hypercalciuria and promote kidney stone formation as well as the optimal level of 25-hydroxyvitamin D (25-OH-D_3_) and appropriate use of vitamin D supplementation in patients with KDT remain controversial.

Considering the above-mentioned arguments, in this study, we aimed to establish the correlation between vitamin D_3_ dose and the occurrence of hypercalciuria/urolithiasis in patients undergoing KDT. In addition, we evaluated the optimal dose to minimize renal complications.

## Materials and methods

### Participants

Overall, 140 patients with intractable childhood epilepsy were started on KDT at a 3:1 lipid to non-lipid ratio in the pediatric neurology department of Severance Children’s Hospital, Seoul, South Korea from January 2016 to December 2019. All patients were referred to the pediatric endocrine department for monitoring the endocrinologic adverse effects of KDT, such as growth retardation, dyslipidemia, multivitamin deficiency, and hypothyroidism. Among these patients, those who maintained KDT for > 3 months and regularly visited the pediatric endocrine department for monitoring were included in this study. Regular outpatient visits were recommended at 1, 3, 6, and 12 months, unless there were medical issues. Patients who already had urolithiasis and received hydrochlorothiazide before KDT, failed to maintain KDT for 3 months, did not take the prescribed calcium and vitamin D_3_ supplementation properly, or did not undergo endocrinological follow-up studies including biochemical laboratory tests, were excluded. Additionally, we excluded patients requiring prolonged hospitalization due to serious illness after KDT initiation, since the changes in their systemic condition and changes in treatment such as AEDs and KDT could significantly alter their clinical aspects. Further, data from patients who started diuretic agents, such as hydrochlorothiazide, were eliminated from that point in time because the excretion of calcium in the urine may be altered in these patients. Finally, 49 patients were included in this study. Among those who continued KDT, 6-month data were obtained from 38 patients and 1-year data were obtained from 22 patients.

The type and dosage of AEDs were not changed significantly during KDT, and the formulations were modified to contain as little carbohydrates as possible. Patients on KDT were supplemented with multivitamins, L-carnitine, calcium, and vitamin D_3_ (cholecalciferol). We used a combination tablet containing 100 mg of calcium and 1,000 IU of vitamin D_3_ per pill for calcium and vitamin D supplementation. Daily doses of calcium and vitamin D_3_ were approximated based on weight: 0.5 tablets (elemental 50 mg of calcium and 500 IU of vitamin D_3_) for a bodyweight up to 10 kg, 1.0 tablet (100 mg of elemental calcium and 1,000 IU of vitamin D_3_) for a bodyweight of 10–20 kg, 1.5 tablets (150 mg of elemental calcium and 1,500 IU of vitamin D_3_) for a bodyweight of 20–40 kg, and 2 tablets (200 mg of elemental calcium and 2,000 IU of vitamin D_3_) for a bodyweight of > 40 kg. If the serum 25-OH-D_3_ level was < 20 ng/mL or > 50 ng/mL, the doses of the calcium and vitamin D_3_ complex were increased or decreased by 0.25 tablets (25 mg of elemental calcium and 250 IU of vitamin D_3_).

The Institutional Review Board of Severance Hospital Clinical Trial Center (subject no. 4-2020-0549) approved this study. Because this was a retrospective study that analyzed only the results obtained during the general course of medical treatment, the need for informed consent was waived. We complied with the Declaration of Helsinki to protect participant rights and personal information.

### Data collection

Patients’ heights and weights were measured at each visit. Further, levels of serum calcium (mg/dL), phosphorus (mg/dL), alkaline phosphatase (mg/dL), 25-OH-D_3_ (ng/mL), parathyroid hormone (PTH, pg/mL), urinary excretion of calcium (Uca, mg/dL), and creatinine (Ucr, mg/dL) in spot urine samples, and urine osmolality were assessed at each visit to identify whether hypercalciuria or any side effects of vitamin D_3_ supplementation had occurred. Serum calcium, phosphate, and alkaline phosphatase levels were measured using Hitachi chemistry autoanalyzer 7600-110 (Hitachi Ltd., Tokyo, Japan) at the central laboratory of Severance Hospital. Serum 25-OH-D_3_ level was determined using a radioimmunoassay (DiaSorin, Inc., Stillwater, MN, United States; intraassay CV < 4.1%, inter-assay CV < 7.0%). The serum PTH concentration was measured at our hospital using a second-generation PTH assay (Elecsys PTH; Roche Diagnostics, Mannheim, Germany) on the Cobas e801 immunoassay analyzer (Roche Diagnostics). Serum osteocalcin level was measured using an electrochemiluminescence immunoassay (Elecsys N-MID Osteocalcin; Roche Diagnostics; intraassay CV < 1.8%, inter-assay CV < 3.3%), and the urinary N-terminal telopeptide was calculated by competitive immunoassay (Vitros™ NTx reagent pack; Ortho-clinical Diagnostics, Inc., Rochester, NY, United States). Urinary calcium excretion (calculated as the ratio of urine calcium level to creatinine level) of the patients was measured by random urine tests using an automated urine chemistry analyzer AU5800 (Beckman Coulter, Fullerton, CA, United States) and LIAISON system (DiaSorin, Saluggia, Italy) at every admission before the initiation of each cycle. The criteria for hypercalciuria were applied differently by age according to the Uca/Ucr ratio (≥ 0.86 for up to 7 months old, ≥ 0.60 for 7–18 months old, ≥ 0.42 for 19 months to 6 years old, and ≥ 0.20 for > 6 years old) (14). The status of the serum 25-OH-D_3_ level was classified as deficient (< 20 ng/mL), insufficient (20–30 ng/mL), and sufficient (> 30 ng/mL) (15).

### Statistical analysis

All statistical analyses were performed using SAS version 9.4 (SAS Inc., Cary, NC, United States) and R package version 3.6.3.^1^ Continuous variables are presented as means and standard deviation (SD). Linear regression analysis was performed to investigate the factors that affect the Uca/Ucr ratio at each visit. To consider the longitudinal structure of data (i.e., four assessment time points), linear mixed-effect models were used to investigate the factors affecting the Uca/Ucr ratio. An autoregressive model (1) correlation structure was assumed among repeated measures of all longitudinal analyses. A logistic regression model was used to investigate factors affecting the occurrence of hypercalciuria at each visit. Moreover, since the occurrence of hypercalciuria was measured at each visit, data were analyzed using the generalized estimating equation model to identify factors affecting the occurrence of hypercalciuria throughout the study period. The results are indicated by odds ratios and confidence intervals. The analysis results of the entire period were adjusted by time effect. To obtain the cut-off value, receiver operating characteristic curve analysis and Youden’s J statistic were used. Statistical significance was set at *p* < 0.05.

## Results

Of the 49 participants enrolled in this study, 31 (63.3%) were boys. All participants were diagnosed with intractable epilepsy, and their seizures began at a mean age of 2.1 ± 2.4 years (range, 0.0–11.7 years). At KDT initiation, the mean age was 4.3 ± 3.2 years (range, 0.3–14.1 years), which was an average of 2.2 ± 2.1 years (0.1–9.5 years) after the onset of seizures (Table 1). The participants received an average of 2.4 ± 1.1 AEDs (range, 0–5 AEDs). The average level of 25-OH-D_3_ was 22.4 ng/mL, with 21 (42.9%) patients being deficient in 25-OH-D_3_, whereas 19 (38.8%) had insufficient levels, and nine (18.4%) had sufficient levels before KDT initiation. One patient had hyperparathyroidism and 25-OH-D_3_ deficiency, and among five patients with hypoparathyroidism, four had insufficient 25-OH-D_3_ levels and one had deficient levels. Although 11 (22.4%) patients already met the definition of hypercalciuria before KDT initiation, they were enrolled in this study because none of them had taken hydrochlorothiazide in accordance with the judgment of the pediatric nephrologists. The patients took an average of 49.9 IU/kg (range, 19.6–102.6 IU/kg) vitamin D_3_ supplementation (Table 1).

**TABLE 1:** Characteristics of children with intractable epilepsy at ketogenic dietary therapy initiation.

	Baseline (*N* = 49)	At 3 months (*N* = 47)	At 6 months (*N* = 38)	At 12 months (*N* = 21)
Sex M: F (n)	31:18	30:17	25:13	14:7
Age (years)	4.3 ± 3.2 (0.3–14.1)	4.6 ± 3.2 (0.6–14.4)	4.7 ± 3.3 (0.8–14.6)	4.4 ± 2.4 (1.3–10.4)
Current AED (n)	2.4 ± 1.1 (0.0–5.0)			
Age at first seizure (year)	2.1 ± 2.4 (0.0–11.7)	2.1 ± 2.4 (0.0–11.7)	2.1 ± 2.5 (0.0–11.7)	1.7 ± 2.0 (0.0–7.5)
Duration of seizure (year)	2.2 ± 2.1 (0.1–9.5)	2.2 ± 2.1 (0.1–9.5)	2.6 ± 2.1 (0.6–10.0)	2.7 ± 1.5 (1.1–7.5)
Height (cm)	100.9 ± 22.2 (64.0–170.1)	103.1 ± 21.9 (65.7–170.0)	105.8 ± 21.7 (71.0–171.0)	103.3 ± 16.8 (78.5–142.5)
Height SDS	−0.03 ± 1.15 (−3.15–1.98)	−0.32 ± 0.91 (−2.44–1.46)	−0.24 ± 0.96 (−1.98–1.66)	−0.42 ± 0.97 (−2.43–1.51)
Weight (kg)	17.5 ± 9.2 (7.1–51.0)	18.2 ± 9.4 (6.9–57.8)	18.9 ± 10.3 (8.7–61.2)	16.8 ± 5.8 (9.0–33.5)
Weight SDS	−0.09 ± 1.31 (−3.20–2.95)	−0.27 ± 1.31 (−3.15–3.47)	−0.36 ± 1.22 (−2.63–2.09)	−0.14 ± 1.02 (−1.90–2.32)
BMI	16.4 ± 2.3 (12.3–24.2)	16.4 ± 2.4 (12.3–22.9)	16.0 ± 2.0 (13.3–20.9)	16.3 ± 1.7 (12.6–20.0)

**Serum variables**				
Calcium (mg/dL)	9.7 ± 0.5 (8.9–10.5)	9.6 ± 0.6 (8.3–11.4)	9.6 ± 0.5 (8.8–10.6)	9.5 ± 0.5 (8.8–10.4)
Phosphorus (mg/dL)	5.3 ± 0.6 (4.1–6.8)	4.7 ± 0.5 (3.1–6.1)	4.8 ± 0.5 (3.8–6.0)	4.8 ± 0.6 (3.7–5.8)
ALP (mg/dL)	231.7 ± 89.2 (68.0–499.0)	186.6 ± 57.6 (66.0–321.0)	196.7 ± 80.6 (52.0–441.0)	205.2 ± 85.4 (97.0–433.0)
PTH (pg/mL)	26.4 ± 12.0 (8.0–75.7)	17.8 ± 7.0 (6.8–33.6)	20.1 ± 8.8 (6.1–42.2)	19.6 ± 5.3 (9.0–28.1)
25-OH-D_3_ (ng/mL)	22.4 ± 9.0 (9.8–49.1)	35.5 ± 9.9 (10.1–58.8)	33.9 ± 9.9 (11.8–55.0)	29.9 ± 8.5 (12.3–48.8)
Deficiency, n (%)	21 (42.9%)	3 (6.1%)	2 (5.4%)	1 (4.5%)
Insufficiency, n (%)	19 (38.8%)	8 (16.3%)	12 (32.4%)	10 (45.5%)
Sufficiency, n (%)	9 (18.4%)	38 (77.6%)	23 (62.2%)	9 (50.0%)
Not checked			1	1

**Urinary excretion**				
Calcium	8.9 ± 9.2 (0.0–39.8)	31.1 ± 22.8 (2.2–95.6)	25.3 ± 21.1 (3.3–83.4)	26.3 ± 17.4 (0.5–59.5)
Creatinine	57.8 ± 38.3 (3.6–178.9)	72.0 ± 52.8 (3.6–264.5)	79.5 ± 69.6 (3.8–399.0)	83.5 ± 57.7 (12.2–244.0)
Uca/Ucr	0.26 ± 0.38 (0.00–1.63)	0.6 ± 0.6 (0.0–2.8)	0.5 ± 0.4 (0.0–2.1)	0.4 ± 0.3 (0.0–1.4)
Hypercalciuria (n, %)	11, 22.4%	27, 57.4%	19, 50.0%	9, 42.9%

Vitamin D_3_ supplementation (IU/kg)		50.8 ± 18.3 (15.4–102.6)	44.5 ± 20.4 (0.0–81.3)	35.1 ± 17.4 (0.0–66.1)

25-OH-D_3_, 25-hydroxyvitamin D; AED, anti-epileptic drugs; ALP, alkaline phosphatase; BMI, body mass index; BSA, body surface area; PTH, parathyroid hormone; SDS, standard deviation score; Uca/Ucr, urinary excretion of calcium/urinary excretion of creatinine ratio.

Three months after KDT initiation with an average vitamin D_3_ supplementation of 50.8 IU/kg, only one patient had hypercalcemia (serum calcium, 11.4 mg/dL; normal range, 8.5–10.5 mg/dL). The patient was administered 55.6 IU/kg of vitamin D_3_ and 100 mg of elemental calcium, and their 25-OH-D_3_ level increased from 30.84 to 46.7 ng/mL and PTH level was low at 7.8°pg/mL. In addition, he had hypercalciuria, with a Uca/Ucr ratio of 2.56. Therefore, we reduced the supplemental doses of vitamin D_3_ and calcium by half (vitamin D_3_, 27.8 IU/kg; elemental calcium, 50 mg/kg). Although his 25-OH-D_3_ level remained similar (43.4 ng/mL) during the follow-up observation, hypercalcemia, and hypercalciuria resolved (Uca/Ucr ratio = 0.19) without any medication. Hyperparathyroidism was not observed in any patient, whereas hypoparathyroidism was identified in 17 (34.7%) patients; however, there was no association between PTH level and Uca/Ucr ratio (Table 2). Hypercalciuria was observed in 27 (55.5%) patients; however, no factors affected Uca/Ucr ratio (Table 2). Moreover, the risk of hypercalciuria decreased as the dose of vitamin D_3_ supplementation increased (odds ratio = 0.950; *p* = 0.014) (Table 3).

**TABLE 2:** Factors affecting the ratio of urinary excretion of calcium (Uca) to urinary excretion of creatinine ratio (Ucr) using longitudinal mixed-effect models.

	Month 3			Month 6			Month 12			Overall		
	β	SE	*P*-value	β	SE	*P*-value	β	SE	*P*-value	β	SE	*P*-value
Sex (ref = M)	0.088	0.188	0.640	0.159	0.100	0.118	0.291	0.140	0.045	–0.119	0.167	0.487
Age at KDT initiation	–0.038	0.028	0.180	–0.034	0.014	**0.024**	–0.036	0.021	0.086	–0.029	0.030	0.349
Seizure onset age	–0.036	0.037	0.333	–0.039	0.020	0.053	–0.035	0.028	0.219	–0.021	0.038	0.589
Height (cm)	–0.006	0.004	0.142	–0.006	0.002	**0.009**	–0.006	0.003	**0.049**	–0.006	0.004	0.159
Weight (kg)	–0.015	0.009	0.128	–0.012	0.005	**0.017**	–0.013	0.007	0.096	–0.014	0.012	0.254
Vitamin D (IU/kg)	–0.002	0.005	0.655	–0.002	0.002	0.352	–0.006	0.003	0.076	–0.002	0.004	0.622
25-OH-D_3_ (ng/mL)	–0.007	0.010	0.486	–0.011	0.005	**0.027**	–0.015	0.007	**0.028**	–0.016	0.009	0.093
PTH	–0.020	0.011	0.0778	–0.004	0.007	0.5772	–0.010	0.009	0.2499	–0.008	0.005	0.1153
Osteocalcin	–0.0006	0.0015	0.7057	–0.0003	0.0005	0.6081	–0.0004	0.0003	0.2393	–0.0003	0.0004	0.4157
NTx	0.0003	0.0002	0.1123	0.00004	0.0002	0.8660	–0.0004	0.0003	0.2856	0.0003	0.0001	0.0080

25-OH-D_3_, 25-hydroxyvitamin D; β, beta coefficient; KDT, ketogenic dietary therapy; SE, standard error; PTH, parathyroid hormone; NTx, N-telopeptide; Uca, urinary excretion of calcium; Ucr, urinary excretion of creatinine; ref, reference; M, male.

Statistically meaningful data are shown in bold.

**TABLE 3:** Odds ratio of factors related to the occurrence of hypercalciuria.

	Month 3		Month 6		Month 12		Overall	
	OR (95% CI)	*P*-value	OR (95% CI)	*P*-value	OR (95% CI)	*P*-value	OR (95% CI)	*P*-value
Sex (ref = M)	1.008 (0.299, 3.403)	0.989	1.745 (0.437, 6.972)	0.431	0.429 (0.057, 3.223)	0.410	1.213 (0.515, 2.857)	0.658
Age at beginning KDT	1.047 (0.869, 1.261)	0.629	0.896 (0.727, 1.105)	0.303	1.187 (0.814, 1.733)	0.374	1.030 (0.869, 1.221)	0.733
Seizure onset age	1.072 (0.831, 1.382)	0.594	0.861 (0.645, 1.148)	0.307	1.318 (0.800, 2.172)	0.279	1.028 (0.814, 1.300)	0.814
Height (cm)	1.012 (0.985, 1.041)	0.379	0.980 (0.950, 1.011)	0.208	1.010 (0.962, 1.060)	0.691	1.004 (0.981, 1.027)	0.756
Weight (kg)	1.009 (0.947, 1.076)	0.779	0.953 (0.878, 1.034)	0.249	1.042 (0.898, 1.209)	0.587	0.998 (0.942, 1.058)	0.952
Vitamin D (IU/kg)	0.950 (0.911, 0.990)	**0.014**	0.956 (0.918, 0.995)	**0.028**	1.005 (0.956, 1.056)	0.857	0.976 (0.954, 0.999)	**0.043**
25-OH-D_3_ (ng/mL)	0.963 (0.901, 1.029)	0.267	0.888 (0.812, 0.971)	**0.010**	0.955 (0.851, 1.072)	0.436	0.945 (0.912, 0.979)	**0.002**

25-OH-D_3_, 25-hydroxyvitamin D; KDT, ketogenic dietary therapy; OR, odds ratio; CI, confidence interval; ref, reference; M, male.

Statistically meaningful data are shown in bold.

**FIGURE 1:**
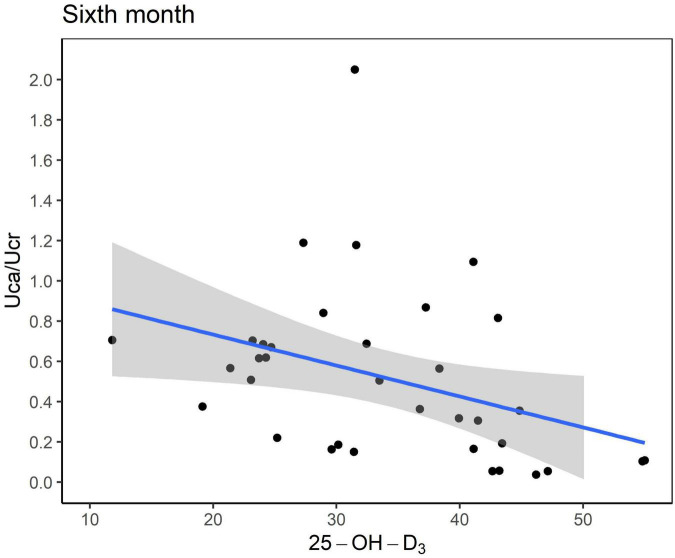
Scatter plot between the urinary excretion of calcium (Uca) to urinary excretion of creatinine (Ucr) ratio and serum vitamin D level (ng/mL). Uca, urinary excretion of calcium; Ucr, urinary excretion of creatinine.

**FIGURE 2:**
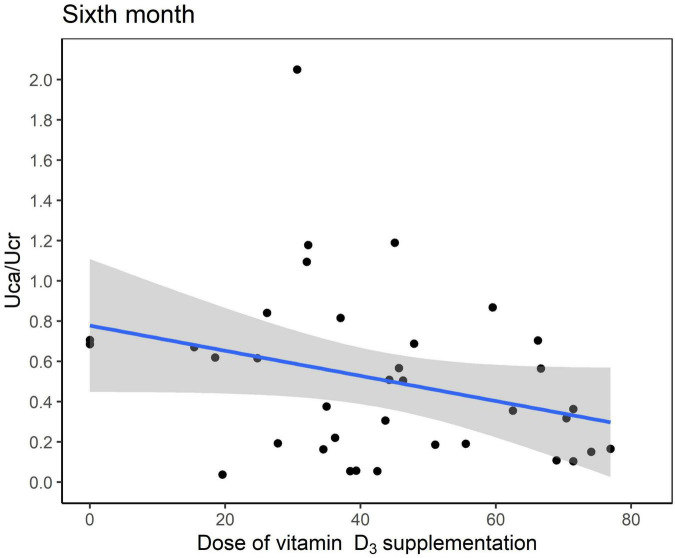
Scatter plot between the urinary excretion of calcium (Uca) to urinary excretion of creatinine (Ucr) ratio and dose of vitamin D_3_ supplementation at 6 months after ketogenic dietary therapy initiation.

**FIGURE 3:**
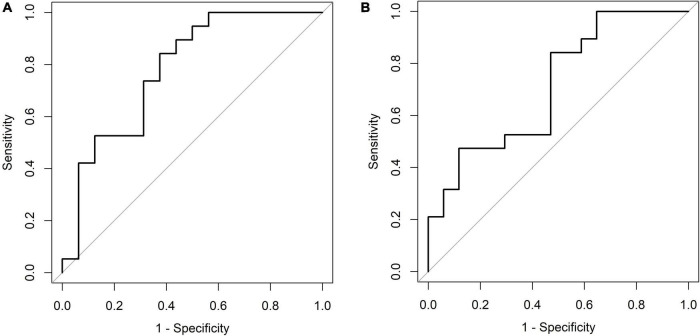
**(A)** Receiver operating characteristic plot [6 months, 25-OH-D_3_ level (ng/mL)]. Cut point: ≤ 39.14, > 39.14. Area under the curve (AUC): 0.7796 (0.6179, 0.9413). **(B)** Receiver operating characteristic plot [6 months, vitamin D_3_ supplementation dose (IU/kg)]. Cut point: ≤ 49.47, > 49.47. Area under the curve (AUC): 0.7121 (0.5399, 0.8843).

Six months after KDT initiation with an average vitamin D_3_ supplementation dose of 44.5 IU/kg, calcium and phosphorus levels of all participants were within the normal range. There were 13 (34.0%) patients with hypoparathyroidism and none with hyperparathyroidism. Hypercalciuria was observed in 19 (50.0%) patients. Age at KDT initiation, height, weight, and 25-OH-D_3_ levels were negatively associated with Uca/Ucr ratio (Figure 1 and Table 2). Additionally, an increased dose of vitamin D_3_ supplementation (odds ratio = 0.956; *p* = 0.028) and a sufficient 25-OH-D_3_ level (odds ratio = 0.888; *p* = 0.010) decreased the risk of hypercalciuria (Figure 2 and Table 3). The optimal level of 25-OH-D_3_, which minimizes the occurrence of hypercalciuria with maximum sensitivity and specificity, was 39.14 ng/mL (Figure 3A), and the optimal dose of vitamin D_3_ supplementation was 49.47 IU/kg (Figure 3B).

One year after KDT initiation with an average vitamin D_3_ supplementation dose of 35.3 IU/kg, calcium and phosphorus levels of all participants were within the normal range, and six (27.3%) patients had hypoparathyroidism. Further, nine patients (40.9%) had hypercalciuria. The dose of vitamin D_3_ supplementation and 25-OH-D_3_ levels did not affect the Uca/Ucr ratio, whereas height and 25-OH-D_3_ levels were negatively associated with Uca/Ucr ratio (Table 2). However, none of these factors increased the risk of hypercalciuria occurrence (Table 3).

In the analysis of the overall follow-up period in which the time variable was corrected through the linear mixed model, the increased dose of vitamin D_3_ supplementation (odds ratio = 0.976; *p* = 0.043) and increased 25-OH-D_3_ level (odds ratio = 0.945; *p* = 0.002) decreased the risk of hypercalciuria, consistent with the trend shown at 3 and 6 months.

Urolithiasis developed in three patients (6.1%): two boys and one girl. Patient 1 was a 3-month-old boy for whom vitamin D_3_ (60.98 IU/kg) was prescribed and discontinued 6 months after KDT initiation because of kidney stone formation. Patient 2 was a 7-year-old girl for whom 45.66 IU/kg of vitamin D_3_ was prescribed. She was diagnosed as having urolithiasis 6 months after KDT initiation; however, vitamin D_3_ supplementation was continued. This was because (1) her 25-OH-D_3_ level was only 21.39 ng/mL, which was only slightly higher than the lower recommended limit of vitamin D_3_, and (2) up to 40–60% of kidney stones in children are reported to be non-calcium-based; considering the lower vitamin D levels, it was unlikely that her urolithiasis was calcium-based (14). Patient 3 was a 19-month-old boy who was diagnosed with urolithiasis 9 months after KDT initiation and vitamin D_3_ supplementation (51.02 IU/kg), even though he did not develop hypercalciuria during the follow-up period. Before KDT initiation, these three patients had neither hypercalciuria nor urolithiasis, and all children with documented stones were first managed medically with increased fluids and urine alkalization using oral potassium citrate to yield a urine pH of 6.5. All three patients reached remission within 2 years with the aid of medical treatment and did not require lithotripsy for their kidney stones. For patients 2 and 3, vitamin D_3_ supplementation was continued for the remission of urolithiasis.

## Discussion

To our best knowledge, this is the first study to assess the relationship between several clinical variables, including vitamin D_3_ dose, serum 25-OH-D_3_ level, and occurrence of hypercalciuria/urolithiasis in pediatric KDT patients. We found that serum 25-OH-D_3_ level and hypercalciuria have an inverse correlation, and as 25-OH-D_3_ level rises by 1.0 ng/mL, Uca/Ucr ratio decreases by 0.011. The optimal serum 25-OH-D_3_ level for preventing hypercalciuria was > 39.1 ng/mL, and the cut-off vitamin D_3_ supplementation dose was > 49.5 IU/kg.

Kidney stone formation is a complex process, which includes urine supersaturation and nucleation, growth, aggregation, and retention of crystals in the kidney (13). KDT can cause kidney stone formation, and the incidence of urolithiasis in children undergoing KDT is 1.4–7% (5, 10, 16). This might be due to hyperuricemia, which increases calcium excretion related to metabolic acidosis, or urine acidification, which results in uric acid supersaturation and decreased urinary citrate concentration (5). According to the “free-particle theory” and “fixed-particle theory,” supersaturated urine is the key process involved in kidney stone formation because the formation and growth of crystals occurs within highly saturated urine (11, 17).

It is unclear whether vitamin D supplementation or high serum 25-OH-D_3_ level increases the risk of hypercalciuria or kidney stone formation. Many physicians are hesitant to treat vitamin D deficiency in patients with kidney stones because of concerns that vitamin D_3_ supplementation increases urinary calcium excretion. This hesitation might be because the most prevalent type of kidney stone is calcium based, and vitamin D increases intestinal calcium absorption and then urinary calcium excretion (18). Calcitriol binds to vitamin D receptors in enterocytes and increases calcium absorption (19). In addition, intestinal calcium absorption is increased in absorptive hypercalciuria (20), and calcitriol serum levels are also correlated with urinary calcium excretion (21). According to a systematic review and meta-analysis, increased circulating calcitriol was associated with kidney stones, and among patients with urolithiasis, circulating 25-OH-D_3_ levels were markedly higher in hypercalciuria than in normocalciuria (22). Therefore, vitamin D is often cited as a risk factor for hypercalciuria and kidney stones (19).

However, several studies have shown that vitamin D supplementation is not associated with urolithiasis. In a prospective study, despite supplementation with high-dose vitamin D_3_ (mean daily dose, 3,440 IU) in healthy controls to maintain 25-OH-D_3_ levels within 30–88 ng/mL for 6 months, no hypercalcemia or hypercalciuria was noted (23). Another study on patients with urolithiasis showed that hypercalciuria or significant changes in urinary calcium excretion did not occur when 50,000 IU of vitamin D_3_ was administered each week, and there was no relation between 25-OH-D_3_ level change and urinary calcium excretion (24). A large systematic review and meta-analysis study found that vitamin D supplementation may increase the risk of hypercalcemia and hypercalciuria, but did not increase the risk of kidney stone formation, regardless of the duration of supplementation, dosage, co-supplementation with calcium, and baseline 25-OH-D_3_ level (12). In a large cohort study, there was also no association found between vitamin D_3_ intake and incidence of kidney stones (25).

Furthermore, despite being controversial, vitamin D deficiency may be a predisposing factor for kidney stone formation. Several studies have shown that vitamin D deficiency is more prevalent in patients with kidney stone formation than in those without (26, 27). There are several hypotheses, as follows: first, secondary hyperparathyroidism caused by vitamin D deficiency can lead to urolithiasis. Second, there are several risk factors shared between vitamin D deficiency and urolithiasis, including obesity and decreased dietary calcium intake. Third, vitamin D deficiency might be responsible for inducing oxidative stress and inflammation in the kidney, which can cause urolithiasis (13).

Vitamin D deficiency causes a decrease in the absorption of dietary calcium, resulting in secondary hyperparathyroidism, which attempts to maintain serum calcium by mobilizing calcium from the bones by increasing osteoclastic activity. These processes decrease bone mineral density. Moreover, hyperparathyroidism increases phosphorus wasting in the kidneys, which results in a low normal or low serum phosphorus level. This results in an inadequate calcium-phosphorus product, causing a mineralization defect in the bones. Consequently, vitamin D deficiency results in osteopenia and osteoporosis (15).

In addition to bone health, vitamin D has various health benefits, as vitamin D receptors exist in most tissues and cells and active vitamin D influences the expression levels of more than 200 genes (28). Vitamin D deficiency causes muscle weakness, whereas increased 25-OH-D_3_ level markedly improves performance speed and proximal muscle strength (29). Further, vitamin D has recently been found to be a key factor in the immune system, as (1) it induces the production of antimicrobial peptides and cytokines, (2) it simulates autophagy for controlling intracellular infections, and (3) vitamin D signaling promotes innate immune response. Thus, vitamin D deficiency is associated with susceptibility toward infections (30). In addition, active vitamin D has biological actions, including angiogenesis, renin production, insulin stimulation, macrophage cathelicidin production, and cellular proliferation inhibition (31). In chronic inflammatory diseases, such as type 2 diabetes and autoimmune diseases, vitamin D is supposed to play an important role in gene regulation (32), and supraphysiological doses of the active form of vitamin D may reduce excessive cell proliferation, even in cancer (31). Furthermore, vitamin D supplementation has been suggested to be potentially preventative against cardiovascular diseases through several mechanisms including upregulation of the renin-angiotensin-aldosterone system, blood pressure increase, and ventricular musculo-hypertrophy (33). In a meta-analysis of eight prospective cohort study, the group with the lower 20% of serum vitamin D levels was associated with increased cardiovascular mortality and all-cause mortality (34). Vitamin D deficiency is also correlated with dyslipidemia (35) and is thought to be more influential in high-fat diets such as KDT.

Given the known benefits of vitamin D in maintaining bone health and its potential benefits for cardiovascular, autoimmune, and neoplastic diseases, and given findings suggesting its safety, active vitamin D supplementation is required in patients undergoing KDT. In addition, we found out that maintaining adequate levels of vitamin D is helpful for hypercalciuria and urolithiasis. Serum 25-OH-D_3_ level and Uca/Ucr ratio showed an inverse correlation during KDT, and although not statistically significant, Uca/Ucr ratio decreased with an increase in the dose of vitamin D_3_ supplementation per weight. In addition, results at 6 months of KDT showed that 25-OH-D_3_ level of < 39.1 ng/mL and inadequate vitamin D supplementation of < 49.5 IU/kg could also increase the risk of hypercalciuria. Therefore, it might be helpful to maintain sufficient serum levels of vitamin D (almost 40 ng/mL) and implement vitamin D supplementation (50 IU/kg) to prevent hypercalciuria.

Although there is no consensus on the optimal serum levels of 25-OH-D_3_, vitamin D deficiency is defined with a 25-OH-D_3_ level of < 20 ng/mL, relative insufficiency with levels between 20 and 29 ng/mL, and sufficient level is ≥ 30 ng/mL. Further, vitamin D poisoning is defined by 25-OH-D_3_ level > 150 ng/mL (15, 28). In a study involving adults, the maximum bone mineral density was achieved when the 25-OH-D_3_ level reached ≥ 40 ng/mL (29). The recommended dose of vitamin D supplementation in children is approximately 400–1,000 IU per day to avoid deficiency and maintain the proper range and 1,000–2,000 IU per day with calcium supplementation for the treatment of vitamin D deficiency (15). According to our findings, the optimal 25-OH-D_3_ level and supplemental doses are similar to those previously recommended by experts. Therefore, although it is necessary to adjust the vitamin D supplementation dose according to the patient’s condition, it is better to actively supplement vitamin D than to hesitate due to concerns about hypercalciuria and urolithiasis.

This study has several limitations. First, it was a retrospective study conducted only with patients who received KDT of 3:1 ratio. Moreover, regular visits were difficult for candidates of this study due to severe neurological disorders and a higher risk of complication compared with normal children. For these reasons, a follow-up duration of > 6 months was difficult for many patients, which was thought to be the reason for the absence of clear statistical association over the entire follow-up period. Statistically significant results were obtained only from data within 6 months of the optimal 25-OH-D_3_ level and appropriate dose of vitamin D supplementation. Second, this study was conducted only on consecutive patients who were referred to the pediatric endocrine department of a single institute, suggesting a distortion in our conclusion owing to the inevitable selection bias. Third, we failed to consider the effects of the anticonvulsants and the supplemental nutrients used by the subjects. AEDs like carbonic anhydrase inhibitors may affect urinary calcium excretion. Herein, nine and seven patients were taking zonisamide and topiramate, respectively, during KDT. However, as these medications are often prescribed for intractable epilepsy, several other patients also used those medications before KDT. As we could not determine the effects of those medications, we did not exclude patients taking them. Though there were no prescription of important supplemental nutrients except multivitamins, personal checks for all the purchased supplements were not possible; hence, we were unable to consider the effects of other supplements.

Nevertheless, this study was meaningful because it gave suggestions for vitamin D supplementation to children on KDT, who are at risk of poor bone health and secondary osteoporosis. Some of the study results showed that contrary to the traditional belief, vitamin D supplementation can help reduce the risk of hypercalciuria during KDT. In addition, we believe that this study can provide a new perspective on the kidney-related side effects that are generally of concern when vitamin D supplementation is implemented. Hypercalciuria and urolithiasis are associated with dietary factors such as intake of fewer fruits and vegetables and more red meat and salt (13, 36), making it difficult to control the variables in a normal population. In this regard, our study has the advantage that it was conducted under the same, controlled dietary conditions. In addition, the results will be applicable to children undergoing KDT.

In conclusion, we recommend that all children on KDT receive 50 IU/kg of daily vitamin D supplementation and maintain a serum 25-OH-D_3_ level of 40 ng/mL to minimize the incidence of hypercalciuria. Further studies with larger numbers of multicenter patients over a longer period of follow-up are required for more evidence and better recommendations.

## Data availability statement

The raw data supporting the conclusions of this article will be made available by the authors, without undue reservation.

## Ethics statement

The studies involving human participants were reviewed and approved by the Institutional Review Board of Severance Hospital Clinical Trial Center. Written informed consent from the participants’ legal guardian/next of kin was not required to participate in this study in accordance with the national legislation and the institutional requirements.

## Author contributions

ML and AK: conceptualization, methodology, and writing—original draft preparation. HL, KS, HSC, JS, and SK: formal analysis, investigation, resources, and data curation. HWC, H-CK, JL, HK, H-SK, and AK: writing—review and editing and supervision. All authors contributed to the article and approved the submitted version.

## Footnotes

1
http://www.R-project.org
